# Gamma Knife Radiosurgery Treatment for Metastatic Melanoma of the Trigeminal Nerve and Brainstem: A Case Report and a Review of the Literature

**DOI:** 10.1155/2013/256962

**Published:** 2013-09-30

**Authors:** Halloran E. Peterson, Erik W. Larson, Robert K. Fairbanks, Wayne T. Lamoreaux, Alexander R. Mackay, Jason A. Call, John J. Demakas, Barton S. Cooke, Christopher M. Lee

**Affiliations:** ^1^Gamma Knife of Spokane, 910 W. 5th Avenue, Suite 102, Spokane, WA 99204, USA; ^2^Cancer Care Northwest, 910 W. 5th Avenue, Suite 102, Spokane, WA 99204, USA; ^3^University of Washington School of Medicine, 1959 NE Pacific Street, Seattle, WA 98185, USA; ^4^MacKay and Meyer MDs, 711 S. Cowley Street, Suite 210, Spokane, WA 99202, USA; ^5^Spokane Brain & Spine, 801 W. 5th Avenue, Suite 210, Spokane, WA 99204, USA

## Abstract

*Objective and Importance*. Brainstem metastases (BSMs) are uncommon but serious complications of some cancers. They cause significant neurological deficit, and options for treatment are limited. Stereotactic radiosurgery (SRS) has been shown to be a safe and effective treatment for BSMs that prolongs survival and can preserve or in some cases improve neurological function. This case illustrates the use of repeated SRS, specifically Gamma Knife radiosurgery (GKRS) for management of a unique brainstem metastasis. *Clinical Presentation*. This patient presented 5 years after the removal of a lentigo maligna melanoma from her left cheek with left sided facial numbness and paresthesias with no reported facial weakness. Initial MRI revealed a mass on the left trigeminal nerve that appeared to be a trigeminal schwannoma. *Intervention*. After only limited response to the first GKRS treatment, a biopsy of the tumor revealed it to be metastatic melanoma, not schwannoma. Over the next two years, the patient would receive 3 more GKRS treatments. These procedures were effective in controlling growth in the treated areas, and the patient has maintained a good quality of life. *Conclusion*. GKRS has proven in this case to be effective in limiting the growth of this metastatic melanoma without acute adverse effects.

## 1. Introduction

Metastases are the most common brain neoplasm, occurring in 10–30% of adult cancer patients. Of these, about 5% occur in the brainstem. Lung, melanoma, renal, breast, and colorectal cancers are the most common primary tumors in these cases [1]. The most common mechanism of metastasis is through hematogenous spread, and distribution of metastases is proportional to the relative blood flow of different areas of the brain [2]. Despite their relative infrequency, brainstem lesions are serious complications because they cause substantial neurological deficit and are usually not surgically resectable [3]. Whole brain radiation therapy (WBRT) and stereotactic radiosurgery (SRS) in combination or alone have therefore become common treatment strategies for brainstem metastases (BSMs). 

Here, we present the case of a patient with a tumor of the trigeminal nerve initially diagnosed as a schwannoma but later discovered to be metastatic melanoma with involvement of the lateral pons through what was most likely microscopic perineural invasion of a lentigo maligna melanoma of the cheek. 

## 2. Case Report

**Figure 1:**
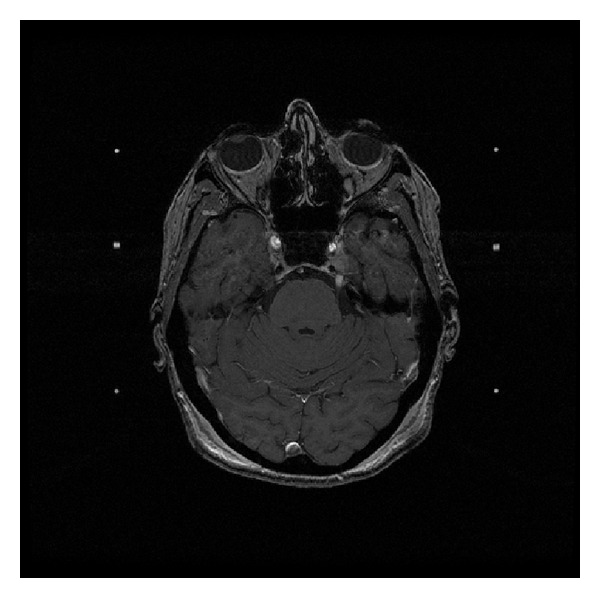
Axial T1 postgadolinium enhanced MRI showing the lesion of the left trigeminal nerve at the time of its original diagnosis as trigeminal schwannoma.

**Figure 2:**
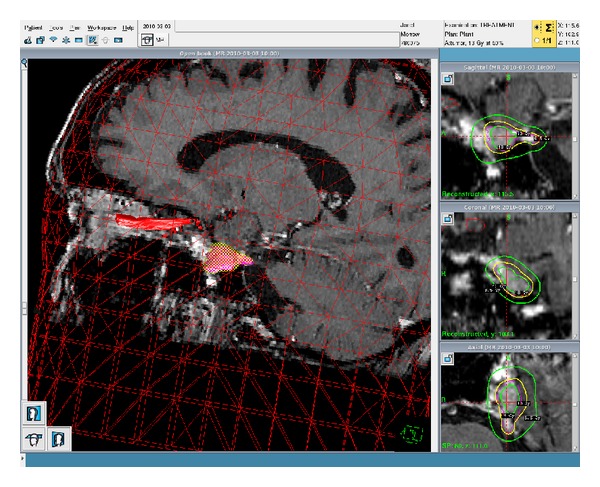
Three-dimensional rendering of the tumor in treatment planning of the patient's first GKRS treatment; the tumor is highlighted and is posteroinferior to the optic nerve which is also highlighted.

The 78-year-old female patient presented initially with one-year history of gradually progressing left facial numbness involving the upper lip, cheek, and forehead accompanied by electric shock-like sensations. Her past medical history was significant for a lentigo maligna melanoma removed 5 years before this initial presentation. An MRI revealed an enhancing mass lesion of the left trigeminal nerve with an appearance consistent with trigeminal schwannoma. The lesion extended from the preganglionic segment into the cavernous sinus. Two months later, another MRI revealed a slight increase in size of the lesion (Figure 1). Three months after her initial presentation, the patient received her first Gamma Knife treatment of 13 Gy at the 50% isodose line (Figure 2). This dose was determined based on standard treatment doses for vestibular schwannoma, a lesion also routinely treated with Gamma Knife. An MRI three months after treatment showed a decrease in size of the mass, and the patient reported a modest decrease in symptoms. 

One year later, an MRI showed interval enlargement of the treated lesion. The tumor had developed a cystic appearance with definite growth at the edges. At this time, the patient reported a worsening of her symptoms to include left oculomotor paresis and diplopia, increased facial weakness and numbness over all three divisions of the trigeminal nerve, and atrophy of the left masseter. Since such aggressive progression would be inconsistent with schwannoma, a PET/CT was ordered to determine if the tumor was of a more malignant variety. PET/CT showed intense glucose uptake by the lesion in question. To determine the specific histologic origin of the tumor and thus optimize treatment, a temporal craniotomy and biopsy were performed. Microscopic examination and immunohistochemical staining of the biopsy indicated that it was metastatic malignant melanoma.

**Figure 3:**
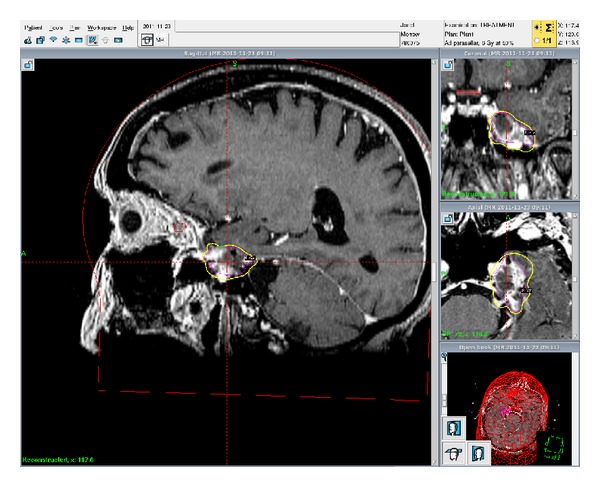
Sagittal treatment planning MRI for the patient's second GKRS treatment showing increased size and the tumor with the Gamma Knife isodose lines in coronal, sagittal, and axial views.

 Based on this revised diagnosis, the patient received a 5-part fractionated Gamma Knife treatment of 6 Gy to the 50% isodose line each time for a total of 30 Gy (Figure 3). The treatments were well tolerated, and during planning of the fifth treatment, it was noted that the tumor appeared smaller than it did at the first of these five treatments. In the four months following, the patient experienced slightly increased sensation in her left face, and MRI showed significant shrinkage of the treated tumor. However, the same MRI also revealed new thickening of the proximal trigeminal nerve adjacent to the brainstem. Due to the excellent response of the tumor to previous radiosurgery, an additional Gamma Knife treatment of 20 Gy to the 50% isodose line was performed. 

The lesion and resulting symptoms remained controlled for approximately 9 months until PET/CT and MRI showed further enhancement at the origin of the trigeminal nerve that had begun to penetrate the left lateral pons as well as further growth distally in the roof of the maxillary sinus. Based on the response to previous radiosurgeries as well as the high quality of the life maintained by the patient, it was determined that another Gamma Knife surgery would be appropriate. Treatment of the pontine lesion was fractionated into three parts of 7 Gy at the 50% isodose line each for a total of 21 Gy. Frame placement allowed only a single treatment of the maxillary lesion at 7 Gy to the 25% isodose line. 

To manage the distal maxillary growth, two options were considered. The mass could be removed surgically by an ENT or treated with stereotactic body radiation therapy (SBRT). After discussion of these options with the patient, it was decided that SBRT would be better tolerated than surgery given the patient's advanced age. 

At the time of this report, the patient was undergoing the planned SBRT to her left maxillary sinus. She has no sensation over her entire left face, and her facial droop is quite significant. To date, she has reported no acute adverse effects from GKRS. 

## 3. Discussion

Brainstem metastases have a poor prognosis with estimated survival between 1 and 6 months [4]. Due to the dense concentration in the brainstem of neural tracts and nuclei essential for normal function, metastases to this area cause severe neurological deficits. These numerous vital structures in close proximity also mean that surgical resection is not usually an option for BSMs. Further, the blood brain barrier limits the utility of chemotherapy agents. 

Since 1999, there have been many studies concluding that SRS is a safe and effective technique for managing BSMs [4–11]. 10 of 20 patients studied by Huang et al. had improvements of their brainstem related neurological deficits after SRS treatment, and no patients died or developed further symptoms from treated tumors. Median survival time (MST) after treatment was 9 months [6]. More recently, Kawabe et al. observed post-SRS survival in combination with neurological deterioration in 200 patients with BSMs. Since only 4–13% of patients with BSMs die of progression of the brainstem lesions themselves (the vast majority dying of systemic disease progression or nonbrainstem intracranial disease), they focused on qualitative survival and SRS effect on neurological function. They found that higher Karnofsky Performance Scale (KPS) scores, single metastases, and well-controlled primary tumors predicted longer survival, while higher KPS and smaller tumor volume predicted increased qualitative survival defined as maintaining a KPS above 70 [8]. The overall MST in the Kawabe study was 6 months, but median survival in patients who were RTOG Recursive Partitioning Analysis (RPA) Class I was 9 months. The MST is likely higher in the Huang study due to patient selection; only 5% of patients had a KPS below 90, while the Kawabe et al. study had 22% below 70.

While SRS has become the primary treatment option for BSMs, radiation tolerance of the brainstem is an important consideration. In an analysis of 279 consecutive radiosurgery procedures, Hong et al. found that at 30 days after procedure, less than 2% of patients experienced serious adverse events requiring hospitalization. 34.1% of these patients experienced acute sequelae, but the vast majority were mild to moderate and included headache, seizures, and fluid retention. Age, diagnosis, or prior radiotherapy was not predictive of sequelae development [12]. Sharma et al. conducted a retrospective study of 38 patients who received Gamma Knife surgery (GKS) to the brainstem to look for incidence of adverse radiation imaging effects (ARIE) on follow-up MRI. They postulate that these imaging changes are mediated by inflammatory processes and found ARIE to correlate with postradiosurgery neurological deficits (*P* = 0.003) including diplopia, facial numbness, dysphagia, dysphonia, weakness, and ataxia. ARIE was observed after exposure of the brainstem to more than 12 Gy [13]. A review of the literature on radiation associated brainstem injury by Mayo et al. showed that the brainstem may be safely treated with 54 Gy using conventional fractionation and 12.5 Gy using SRS [14]. Given these considerations, conformal treatment systems with sharp radiation fall-off should be used where possible to protect healthy brainstem tissue. 

Studies exist comparing the efficacy of WBRT, SRS, or combinations of the two to treat brain metastases; however, specific data on BSMs is limited. A Cochrane review by Patil et al. showed improved performance status in terms of KPS and better local control (HR 0.27; 95% CI 0.14 to 0.52) but no overall survival benefit from both treatments over WBRT alone. However, RPA class I patients and those with only one metastasis did survive longer with combined treatment [15]. Similar results have been found when comparing WBRT and SRS to SRS alone. In a randomized study by Aoyama et al., combination treatment did not confer prolonged survival over SRS alone but did reduce recurrence of targeted tumors as well as distant relapses within the brain requiring salvage treatment (*P* < 0.001) [3]. The primary disadvantage of WBRT is its negative effects on neurological function in long-surviving patients; therefore, the goal of controlling micrometastases must be balanced with considerations of patient quality of life. Chang et al. demonstrated that four months after treatment, patients who receive WBRT and SRS are at a greater risk of decline in memory and learning (mean posterior probability of decline = 52%) than those treated with SRS alone (mean posterior probability of decline = 24%) [16]. While this work by Chang et al. may be limited by its smaller sample size, results of a recent phase III trial of adjuvant WBRT versus observation following surgery or radiosurgery for BMs show a decline in quality of life in the treatment arm indicating that SRS alone is likely favorable in terms of maintaining function [17].

## 4. Conclusion

This is a unique case for a number of reasons, and to our knowledge there are no similar reports. Our patient has an unusual metastatic melanoma masquerading as a trigeminal schwannoma in a critical area that has been managed well with repeat GKRS treatments even after its invasion of the brainstem. This report is part of a growing body of evidence showing that SRS is effective and safe for palliation in the case of BSMs.
